# Effects of Physical and Cognitive Training on Falls and Concern About Falling in Older Adults: Results From a Randomized Controlled Trial

**DOI:** 10.1093/gerona/glab375

**Published:** 2021-12-15

**Authors:** Katri M Turunen, Anna Tirkkonen, Tiina Savikangas, Tuomo Hänninen, Markku Alen, Roger A Fielding, Miia Kivipelto, Anna Stigsdotter Neely, Timo Törmäkangas, Sarianna Sipilä

**Affiliations:** Gerontology Research Center and Faculty of Sport and Health Sciences, University of Jyväskylä, Jyväskylä, Finland; Seinäjoki University of Applied Sciences, School of Health Care and Social Work, Seinäjoki, Finland; Gerontology Research Center and Faculty of Sport and Health Sciences, University of Jyväskylä, Jyväskylä, Finland; Gerontology Research Center and Faculty of Sport and Health Sciences, University of Jyväskylä, Jyväskylä, Finland; NeuroCenter, Neurology, Kuopio University Hospital, Kuopio, Finland; Department of Medical Rehabilitation, Oulu University Hospital, Oulu, Finland; Nutrition, Exercise Physiology, and Sarcopenia Laboratory, Jean Mayer USDA Human Nutrition Research Center on Aging, Tufts University, Boston, Massachusetts, USA; Public Health Promotion Unit, Finnish Institute for Health and Welfare, Helsinki, Finland; Division of Clinical Geriatrics, Center for Alzheimer Research, NVS, Karolinska Institutet, Stockholm, Sweden; Department of Social and Psychological Studies, Karlstad University, Karlstad, Sweden; Department of Psychology, Umeå University, Umeå, Sweden; Gerontology Research Center and Faculty of Sport and Health Sciences, University of Jyväskylä, Jyväskylä, Finland; Gerontology Research Center and Faculty of Sport and Health Sciences, University of Jyväskylä, Jyväskylä, Finland

**Keywords:** Executive functions, Exercise, Fall prevention, Follow-up, Intervention

## Abstract

**Background:**

The aim of this study is to investigate whether combined cognitive and physical training provides additional benefits to fall prevention when compared with physical training (PT) alone in older adults.

**Methods:**

This is a prespecified secondary analysis of a single-blind, randomized controlled trial involving community-dwelling men and women aged 70–85 years who did not meet the physical activity guidelines. The participants were randomized into combined physical and cognitive training (PTCT, *n* = 155) and PT (*n* = 159) groups. PT included supervised and home-based physical exercises following the physical activity recommendations. PTCT included PT and computer-based cognitive training. The outcome was the rate of falls over the 12-month intervention (PTCT, *n* = 151 and PT, *n* = 155) and 12-month postintervention follow-up (PTCT, *n* = 143 and PT, *n* = 148). Falls were ascertained from monthly diaries. Exploratory outcomes included the rate of injurious falls, faller/recurrent faller/fall-related fracture status, and concern about falling.

**Results:**

Estimated incidence rates of falls per person-year were 0.8 (95% confidence interval [CI] 0.7–1.1) in the PTCT and 1.1 (95% CI 0.9–1.3) in the PT during the intervention and 0.8 (95% CI 0.7–1.0) versus 1.0 (95% CI 0.8–1.1), respectively, during the postintervention follow-up. There was no significant difference in the rate of falls during the intervention (incidence rate ratio [IRR] = 0.78; 95% CI 0.56–1.10, *p* = .152) or in the follow-up (IRR = 0.83; 95% CI 0.59–1.15, *p* = .263). No significant between-group differences were observed in any exploratory outcomes.

**Conclusion:**

A yearlong PTCT intervention did not result in a significantly lower rate of falls or concern about falling than PT alone in older community-dwelling adults.

**Clinical Trial Registration:**

ISRCTN52388040

## Background

Falls are major contributors to injuries and death in older adults (1, 2). Approximately 1 in 4 adults aged older than 65 years living in the community will experience a fall each year (3, 4), and 20%–30% of falls cause a serious injury, such as a fracture or head injury (5). Concern about falling is widely recognized as a related but distinct disabling problem, affecting about 2 out of 3 fallers but also up to half of older adults without a previous fall history (6, 7).

Physical exercise is the single most effective intervention in community-dwelling older people, with up to 34% of falls being prevented by well-designed exercise programs that include balance, functional, and strength training (8). Moreover, to a limited extent, exercise reduces concern about falling immediately after the exercise intervention (9). Despite the strong evidence from efficacy trials, the prevention of falls remains suboptimal; consequently, the burden related to falls and concern about falling (economic burden for societies and the human cost, including pain, distress, disability, and loss of quality of life) has continued to rise in Western countries (10). Therefore, it is crucial to find strategies that can enhance the beneficial effects of physical training (PT).

Few fall prevention programs explicitly address the important risk factors for falls and impaired cognition, such as executive functions. Executive functions are needed to allow for fall-free gait in dual-task situations (eg, talking to a companion during walking, reading a street sign, walking on an uneven surface, or planning ahead) and while inhibiting the response to potential distractions (eg, traffic) (11–13). Executive functions are amenable to training (14) and may be key when developing more optimal fall prevention programs. The growing number of randomized controlled trials shows that a training program targeting both physical and cognitive risk factors for falls can promote executive functions (15, 16) and may improve balance control (17) when compared with PT alone. However, executive functions and balance control are indirect measures related to fall prevention, and more research is needed to investigate the direct effects of combined training on falls and fall injuries. Hence, the current study investigates whether the combination of cognitive and physical training would provide additional value in terms of fall prevention and a reduction in the concern about falling compared with PT alone among older adults.

## Method

### Study Design

The present study is a prespecified secondary outcome analysis of a parallel-group, assessor-blind, randomized clinical trial with a 12-month follow-up (the “Promoting Safe Walking Among Older People: Physical and Cognitive Training Intervention Among Older Community-dwelling Sedentary Men and Women,” or the PASSWORD study) conducted in the city of Jyväskylä, Finland. Details of the trial design, recruitment, interventions (18), and primary findings (gait speed and executive function) have been reported previously (15). The trial was registered before recruitment of the participants (http://www.isrctn.com/ISRCTN52388040). Ethical approval was obtained from the Ethical Committee of the Central Finland Health Care District (14/12/2016, ref: 11/2016). All the participants provided written informed consent.

### Participants

**Figure 1.**
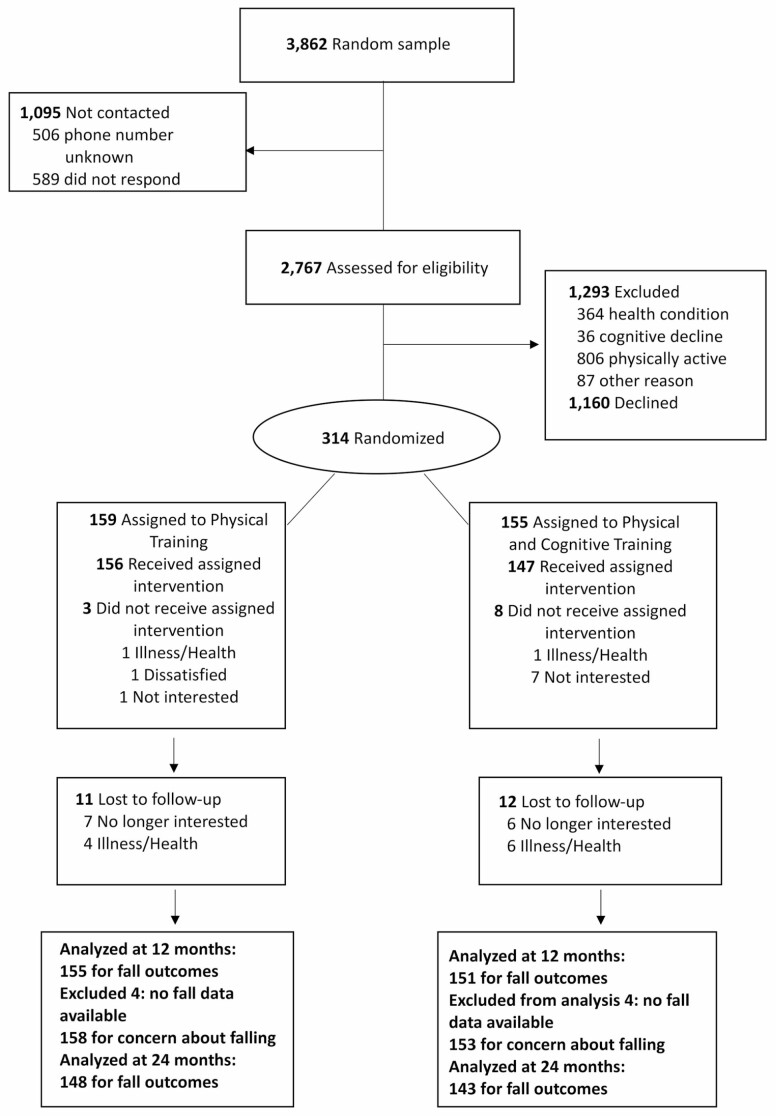
Flow chart of the study.

The participants were community-dwelling adults who were randomly selected from Finland’s Population Information System, which is administered by the Population Register Center. A letter containing information about the study was sent, and interviews were conducted by phone to screen for inclusion and exclusion criteria related to walking, physical activity, and major chronic diseases. Those older adults who fulfilled the inclusion criteria were willing to participate and did not report any exclusion criteria were invited into the clinical examinations. The clinical exclusion criteria were assessed, and the health status was confirmed before the baseline assessments. A flow chart is presented in Figure 1.

Eligible participants were community-dwelling adults aged 70–85 years and living in the city of Jyväskylä, Finland, who did not meet the physical activity guidelines (less than 150 minutes of moderate-intensity aerobic activity in bouts of at least 10 minutes per week and no regular resistance training) (19). Additional inclusion criteria were being able to walk 500 m without assistance and a Mini-Mental State Examination (MMSE) test score of 24 or higher. We excluded older adults suffering from severe chronic or progressive disease, severe musculoskeletal problems, depressive mood (Geriatric Depression Scale [GDS]-15 >10 points), and who did not have the resources to commit to the study (according to the participants themselves or assessments by a physician and the primary investigators), risk level use of alcohol (>7 units of alcohol per week for women and >14 for men), or any other contraindications for PT (18).

### Randomization and Blinding

The participants were randomly assigned in a 1:1 ratio to receive either the physical and cognitive training (PTCT) or PT alone intervention. Randomization was stratified by age (70–74, 75–79, 80–85) and sex; blocks of varying sizes (2 or 4) were used. A computer-generated random number schedule was developed by a statistician, and randomization was performed by a researcher not participating in the data collection process. Assessors collecting the data were blinded to group allocation, and the participants were asked not to disclose their study group to the personnel collecting the data.

### Interventions

The interventions have been described in detail previously (15, 18). Briefly, the interventions lasted for 12 months and started with introductory seminars, including a motivational lecture on physical activity. The PTCT participants also attended an introductory seminar that included detailed information on the cognitive training (CT) portion of their intervention. The interventions included supervised training sessions and home exercises. The PT intervention was adapted from the physical activity guidelines for older adults (19), our earlier studies (20, 21), and the LIFE study (22). It included progressive aerobic, resistance, and balance training. The participants attended twice a week in supervised sessions: once for walking and dynamic balance training and once for resistance and balance training. Walking sessions began with a short walk at a self-selected speed, and dynamic balance exercises followed with continuous walking for 10–20 minutes at a target intensity of “somewhat hard” to “hard.” Resistance training took place in senior gyms equipped with resistance training machines utilizing air pressure technology and Smart Card/Smart Touch Software. Each session started with a 10-minute warm-up and balance exercises, which was followed by 8–9 resistance exercises for the lower body, trunk, and upper body muscles. Five to six different training periods with variations in training specificity, volume, and intensity were used to maintain physiological responses to training. In addition, the training load was further adjusted according to 6 repetition maximum tests that were performed 3 times during the intervention.

The progressive home exercise program included strengthening exercises for the lower limb muscles, balance exercises, and stretching for major muscle groups and was performed 2–3 times per week. In the strengthening exercises, the workload increased with resistance bands. In the standing balance exercises, the level of challenge was increased by reducing hand, base, and vision support. The participants were also advised to accumulate moderate aerobic activity, totaling 150 minutes per week in bouts of at least 10 minutes.

The CT targeted executive functions (inhibition, shifting, and updating of working memory) and was based on the unity/diversity model of executive functions by Miyake and Friedman (23). The CT utilized a web-based, in-house-developed computer program modified from the program used in the FINGER study (24). During the training sessions, which each ran for approximately 20 minutes, different cognitive tasks were practiced. The target training frequency was 3–4 times a week. Those who lacked access to a computer at home had the possibility of attending supervised sessions at least once a week, with the possibility of training in one of 10 locations, with a peer tutor provided by the city of Jyväskylä (in libraries, sheltered accommodation, etc.).

Adherence to the training programs has been reported previously (15). Shortly, 65% of the participants in the PT and 72% of those in the PTCT group attended the supervised PT sessions weekly. The CT was performed on average 1.9 times per week.

### Outcomes

The primary outcome of walking speed and one of the secondary outcomes, executive function, of the PASSWORD study have been reported elsewhere (15). The current paper reports the secondary fall-related outcomes: the overall number of falls over the 12-month intervention and 12-month postintervention follow-up. A fall was defined according to the internationally accepted definition: an unexpected event in which the person comes to rest on the ground, floor, or lower level without an overwhelming extrinsic cause (25). As an exploratory outcome, we report the rate of self-reported injurious falls. Injurious falls were defined as falls that resulted in contact with health care services because of injury. Falls were monitored by calendars that were returned monthly. For each fall, detailed information on its location, injuries, and need for care because of the fall was reported. A research coordinator contacted the participants if they did not return their monthly calendar. Falls were also examined using the proportion of fallers (1 or more falls), recurrent fallers (2 or more falls), and fall-related fractures.

Concern about falling was measured with the Falls Efficacy Scale International (FES-I; 26), which was given to the participants by a research assistant. The questionnaire comprises 16 items assessing, for example, walking on slippery, uneven, or sloping surfaces, visiting friends or relatives, or going to a social event. Concerns about falling when carrying out each activity were assessed on a 4-point scale (ranging from 1 = not at all concerned to 4 = very concerned). The total FES-I score ranges from 16 to 64. The higher the score, the greater the concern about falling. The internal consistency and the test–retest reliability for the FES-I have been shown to be high (26). Concern about falling was measured at baseline and at 6 and 12 months.

### Background Characteristics

Trained research staff were blinded to group allocation and performed all the measurements at baseline. Body height (m) and weight (kg) were measured, and body mass index (kg/m^2^) was calculated. Highest education was self-reported. Education was categorized as low (primary school or less), medium (middle school, folk high school, vocational school, or secondary school), or high (high school diploma or university degree). Self-rated health was reported on a 5-point scale from very good to very poor and dichotomized (very good/good and average/poor). Cognition was measured using the Consortium to Establish a Registry for Alzheimer’s Disease (CERAD) total score (range 0–100), which involves the following subtests: Category Verbal Fluency, Modified Boston Naming Test, MMSE, Word List Memory, and Constructional Praxis. Higher scores indicate better performance. Executive functions were assessed using the Stroop Color–Word Test, which measures response inhibition by deliberate overriding of dominant responses (27).

Mood was assessed using the GDS (range 0–15), with higher scores indicating increased symptomatology (28). Clinical health data were based on self-reports and data collected from the National Health Service integrated patient information system and from a clinical examination. The use of psychotropic drugs, including opiates, benzodiazepines, anticholinergic agents, dopaminergic agents, and antidepressants, was documented according to the anatomical therapeutic chemical classification. Systolic blood pressure and diastolic blood pressure were measured using an aneroid sphygmomanometer, here by following a standardized protocol. Orthostatic hypotension was defined by a drop in blood pressure of at least 20 mmHg for systolic blood pressure or at least 10 mmHg for diastolic blood pressure within 2 minutes of standing up (29). The visual acuity test measured the accuracy of distance vision using the E chart or C chart, both eyes together (with spectacles if the individual wore them), and reported as best-corrected visual acuity (visus).

Physical activity was measured with a hip-worn tri-axial accelerometer (UKK RM42, UKK, Tampere, Finland) for 7 days and analyzed, as reported by Savikangas et al. (30). Physical performance was measured using the Short Physical Performance Battery (31). Information on falls the year before baseline was collected retrospectively by a structured questionnaire. The questions (2 questions answered separately) were as follows: “How many times have you fallen indoors/outdoors during the previous year?” The response options were 1 = none, 2 = once, 3 = 2–4 times, 4 = 5–7 times, and 5 = 8 times or more. For the analyses, the participants who reported ≥1 falls indoors or outdoors were coded as “fallers”; and those who reported ≥2 falls were defined as “recurrent fallers.” In addition, the participants were asked whether they were injured and needed care because of a fall (yes–no). The participants who responded yes were coded as having an injurious fall during the previous year.

### Statistical Analysis

The sample size (*n* = 310) was determined for the primary outcome, walking speed, of the PASSWORD (18). For an additional a priori power analysis for the falls rate, we were unable to find earlier publications with similar design and outcome as in our study. Therefore, we used information from a previously published study from Finland including physical exercise and nonexercise groups (32) to assess the fall rate due to the PT intervention. As we knew that among healthy older adults, executive functions are associated with falls (33) we expected that training executive functions in addition to PT would result in greater benefit in terms of falls rate than physical exercise alone. Thus, at 80% power favoring the PTCT over PT, an assumed drop-out rate of 15%, and 1.2 falls/person-year for participants with physical exercise only, it was expected that it would be possible to detect a difference of 27% in the fall rate between the groups (ie, incidence rate ratio [IRR] 0.73).

All analyses were performed according to the intention-to-treat principle. The baseline characteristics were summarized as means (standard deviations) or frequencies (percentages). Those participants with missing fall data were compared with those who provided at least some prospective fall data using a *t*-test or chi-square test. The incidence rates for falls were calculated in relation to person-years using the number of observation days. The main analysis evaluated the between-group differences in the number of falls over the 12-month intervention and over the 12-month follow-up using a generalized linear model’s negative binomial regression. A negative binomial regression analysis is an extension to the Poisson model that accommodates overdispersion (the variance exceeds the mean) (34). In addition, the between-group differences in the rate of injurious falls during the 12-month intervention and subsequent 12-month follow-up were also modeled using a negative binomial regression. A modified Poisson regression was used to calculate the relative risk of faller, recurrent faller, and fall-related fracture status.

As explorative analysis, we conducted also subanalyses for prespecified groups. They were conducted using the interaction terms (Group × Characteristic) in negative binomial regression models; these assessed whether the PTCT had a differential effect on fall rate in terms of age (70–74, 75–79, and 80–85 years), sex, baseline cognition (CERAD total score <69 is low and ≥69 is high), and level of compliance to the intervention (the high compliance subgroup participated in at least 50% of the supervised walking/dynamic balance sessions and in at least 50% of the resistance/balance training sessions; in the PTCT group, the high compliance subgroup also performed CT at least twice a week).

The effect of the interventions on concern about falling was analyzed using generalized estimating equation models with a Group × Time interaction term. Significance level was set at 0.05. All analyses were conducted using IBM SPSS Statistics 26 (SPSS Inc., Armonk, NY).

## Results

Potential participants (*n* = 2 767) were screened between January 2017 and March 2018. Of these, 314 were randomized to the PTCT (*n* = 155) and PT (*n* = 159) groups. The flow of the participants through the study is described in Figure 1. Eight participants (3% of total sample, 4 from both groups) did not provide any data related to falls after randomization. They did not differ statistically significantly from those who had prospective fall data in terms of age (*p* = .081), sex (*p* = .337), history of fall (*p* = .556), cognition (*p* = .853), mood (*p* = .375), concern about falling (*p* = .234), physical performance (*p* = .058), self-rated health (*p* = .226), or presence of long-term pain (*p* = .888).

The demographic and baseline characteristics of the participants in the 2 groups were similar at baseline (Table 1). Across the groups, the mean age was 74.5 years (*SD* 3.8), 60% were women, 10% (*n* = 30) had an injurious fall during the previous year, and 19% (*n* = 61) had 2 or more falls during the previous year.

**Table 1.** Baseline Characteristics of the Participants by Physical and Cognitive Training (PTCT) and Physical Training (PT) Groups

	PTCT (*n* = 155)	PT (*n* = 159)
Age, mean (*SD*), years	74.4 (3.9)	74.5 (3.7)
Women no. (%)	96 (62)	92 (58)
Body mass index, mean (*SD*), kg/m^2^	28.0 (4.9)	27.9 (4.5)
Education, no. (%)		
Low	23 (15)	25 (16)
Medium	94 (61)	106 (67)
High	38 (25)	28 (18)
MMSE, mean (*SD*)*	27.9 (1.4)	27.4 (1.5)
Stroop effect, mean (*SD*)†	45.2 (20.6)	48.2 (28.7)
SPPB, mean (*SD*)‡	10.2 (1.5)	10.1 (1.6)
Physical activity; accelerometer, min/day mean (*SD*)§		
Sedentary time (<0.0167 g)	604 (86)	601 (80)
Light-intensity activity (≥0.0167 to <0.091 g)	215 (65)	206 (67)
Moderate-to-vigorous-intensity activity (≥0.091)	32 (19)	33 (21)
Moderate-to-vigorous intensity activity in bouts of ≥10 min, min/week, mean (*SD*)	80 (83)	86 (88)
Self-rated health, no. (%)		
Very good/good	73 (47)	68 (43)
Average/poor	82 (53)	91 (57)
GDS score‖		
Mean (*SD*)	1.4 (1.4)	1.8 (1.9)
≥5, no. (%)	7 (5)	14 (9)
Chronic conditions, no. (%)		
Musculoskeletal diseases¶	64 (41)	62 (39)
Metabolic diseases#	101 (65)	117 (74)
Cardiovascular diseases**	46 (30)	49 (31)
Pulmonary diseases††	26 (17)	17 (11)
Mental health diseases‡‡	5 (3)	8 (5)
Neurologic diseases§§	8 (5)	6 (4)
Use of psychotropic, no (%)	24 (15)	21 (13)
Blood pressure, mmHg, mean (*SD*)		
Systolic	148 (19)	153 (20)
Diastolic	78 (9)	79 (10)
Orthostatic hypotension‖‖, no (%)	13 (8)	13 (8)
Visual acuity¶¶	0.80 (0.18)	0.70 (0.18)
Recurrent falls in the past year, no (%)	24 (16)	37 (23)

*Mini-Mental State Examination, total score, range 0–30, higher score indicates better performance.

^†^Stroop incongruent–Stroop neutral in seconds, lower time indicates better performance.

^‡^Short Physical Performance Battery, total score, range 0–12, higher score indicates better performance.

^§^Mean amplitude deviation.

^‖^Geriatric Depression Scale, range 0–15, <5 points indicates normal mood.

^¶^Including arthrosis, endoprosthesis, osteoporosis, back diseases, joint pain, conditions causing pain in the neck and upper extremities, muscular dystrophy, hernia; and inflammatory diseases including rheumatoid diseases, arthritis, psoriatic arthritis, fibromyalgia, polymyalgia, and gout.

^#^Including type 2 diabetes, hypertension, hypercholesterolemia, and other lipid storage disorders.

^**^Including myocardial infarction, stroke, intracranial hemorrhage, coronary artery disease, transient ischemic attack, peripheral arterial disease, intermittent claudication, arrhythmias, heart defect, heart failure, and pacemaker.

^††^Including chronic obstructive pulmonary disease, asthma, pulmonary fibrosis, and bronchiectasis.

^‡‡^Including depression, stress, bipolar disorder, disorientation, and adjustment disorder.

^§§^Including poliomyelitis, migraine, epilepsy, Parkinson’s disease, peripheral neurological diseases, and polyneuropathy.

^‖‖^Defined by a drop in blood pressure of at least 20 mmHg for systolic blood pressure or at least 10 mmHg for diastolic blood pressure within 2 minutes of standing up.

^¶¶^Visus, good = visus at least 1.00, clearly diminished = visus ≤0.50.

### Fall Outcomes

During the 12-month intervention, with a mean follow-up of 350 (47) days, 132 falls occurred among 75 fallers in the PTCT group versus 172 falls among 79 fallers in the PT alone group. The fall rate incidence during the intervention was 0.8 (95% CI 0.7–1.1) per person-years in the PTCT group and 1.1 (0.9–1.3) per person-years in the PT group. The 22% difference in fall rate in the PTCT group compared with the PT group was not statistically significant (IRR = 0.78; 95% CI 0.56–1.10, *p* = .152).

During the 12-month postintervention follow-up (a mean follow-up of 377 ± 20 days), 117 falls were recorded among 64 fallers in the PTCT group versus 148 falls among 62 fallers in the PT group. There were no significant differences between groups in the rate of falls (IRR = 0.83; 95% CI 0.59–1.15, *p* = .263 and IRR = 1.49; 95% CI 0.72–3.06, *p* = .279). There were no significant differences between the PTCT and PT groups in the proportion of fallers, recurrent fallers, or fall-related fractures over the 1-year intervention or 1-year postintervention follow-up (Table 2).

**Table 2.** Fall Outcomes (*n* = 306) Over 12-Month Intervention and 12-Month Postintervention Follow-Up

	PTCT (*n*=151)		PT (*n*=155)		Regression Model, PTCT vs PT		
During 12-Month Intervention	IR	95% CI	IR	95% CI	IRR	95% CI*	p
IR of all falls per person-year	0.83	0.65–1.07	1.06	0.85–1.34	0.78	0.56–1.10	.152
IR of injurious falls per person-year	0.10	0.06**–**0.17	0.09	0.05–0.15	1.20	0.55–2.60	.652
	*n* (%)				Coefficient	95% CI†	
Faller	75(49.7)		79(51.0)		0.97	0.78–1.22	.813
Recurrent faller	33(21.9)		33(21.3)		1.04	0.68–1.59	.869
Fall-related fracture	3(2.0)		8(5.2)		0.39	0.11–1.41	.152
	PTCT (*n* = 143)		PT (*n* = 148)				
During 12-Month Follow-Up	IR	95% CI	IR	95% CI	IRR	95% CI*	
IR of all falls per person-year	0.80	0.66–0.95	0.97	0.82–1.13	0.83	0.59–1.15	.263
IR of injurious falls per person-year	0.14	0.09-0.21	0.09	0.05-0.15	1.49	0.72–3.06	.279
	*n* (%)				Coefficient	95% CI†	
Faller	64(44.8)		62(41.9)		1.08	0.83–1.40	.581
Recurrent faller	26(22.2)		22(17.3)		1.26	0.76–2.10	.366
Fall-related fracture	6(4.2)		4(2.7)		1.56	0.45–4.76	.482

*Notes:* IR = incidence rate; IRR = incidence rate ratio; PTCT = physical and cognitive training; PT = physical training.

*From negative binomial regression analyses.

^†^From modified Poisson regression analyses.

In the prespecified subgroup analyses for the fall rate, the interaction terms (Group × Characteristics) were not significant for age, sex, baseline cognition, and compliance with the training during the intervention and postintervention follow-up. In other words, no statistically significant differences in the intervention effects were found based on sex, age, baseline cognition, or compliance to the training. Although not statistically significant, men in the PTCT group tended to have a lower fall rate than men in PT during the 1-year follow-up (*p* = .079; Table 3). Table 4 presents the mean scores of FES-I and statistics over time. No statistically significant difference was observed in changes of FES-I between the groups (Group × Time interaction, *p* = .688). Both the PTCT and PT groups reduced their concern about falling over the 1-year intervention (on average −0.78 points; CI −1.36 to −0.20, *p* = .007): the average reduction was 3% in the PTCT group and 4% in the PT group.

**Table 3.** Incidence Rates of Falls per Person-Year During 1-Year Intervention and 1-Year Follow-Up According to Subgroups

Subgroup		During 12-Month Intervention					During 12-Month Follow-Up			
	n	≥1 fall *n* (%)*	IR	Interaction (95% CI)†	p	n	≥1 fall *n* (%)*	IR	Interaction (95% CI)†	p
Sex										
PTCT men	57	25 (44)	0.98	0.79 (0.41–1.52)	.479	53	19 (36)	0.93	0.54 (0.27–1.07)	.079
PT men	67	31 (46)	1.41			64	28 (44)	1.44		
PTCT women	94	49 (52)	0.89			90	45 (50)	0.73		
PT women	88	47 (53)	0.97			84	34 (41)	0.61		
Age (years)										
PTCT 70–74	96	50 (52)	0.90	0.56 (0.17–1.88)	.350	89	43 (48)	0.87	0.61 (0.15–2.52)	.498
PT 70–74	93	47 (51)	1.18			91	32 (35)	0.87		
PTCT 75–79	41	16 (39)	0.95	0.51 (0.14–1.84)	.305	40	17 (43)	0.71	0.32 (0.74–1.41)	.133
PT 75–79	47	24 (51)	1.27			43	24 (56)	1.36		
PTCT 80–85	14	8 (57)	1.08			14	4 (29)	0.64		
PT 80–85 years	15	7 (47)	0.68			14	6 (43)	0.43		
Cognition										
PTCT high	140	65 (47)	0.89	0.88 (0.30–2.55)	.808	135	58 (45)	0.81	1.15 (0.35–3.78)	.821
PT high	137	68 (49)	1.14			129	51 (38)	0.97		
PTCT low	14	9 (64)	1.23			14	6 (43)	0.69		
PT low	14	9 (64)	1.31			12	10 (83)	0.96		
Compliance										
PTCT high	112	58 (52)	0.95	0.62 (0.30–1.24)	.172	106	49 (46)	0.75	1.31 (0.63–2.72)	.477
PT high	103	46 (45)	1.02			101	42 (42)	0.99		
PTCT low	39	16 (41)	0.81			37	15 (41)	0.93		
PT low	52	32 (62)	1.43			47	20 (43)	0.92		

*Notes:* IR = incidence rate; PTCT = physical and cognitive training; PT = physical training.

*Number of participants (proportion) who fell at least once.

^†^From negative binomial regression analyses.

**Table 4.** Concern About Falling (FES-I, Score) at Baseline and After 6 and 12 Months of Physical and Cognitive Training (PTCT) or Physical Training Alone (PT)

Outcome	PTCT			PT			Group ×Time Interaction (PTCT–PT)	
	Mean (*SE*)	Difference (95% CI)	n	Mean (*SE*)	Difference (95% CI)	n	Difference (95% CI)	p
Baseline	22.34 (0.39)		153	22.62(0.47)		158		
6 months	21.92 (0.45)	−0.42 (−1.22 to 0.37)	147	21.39 (0.44)	−1.23 (−1.94 to −0.52)	146	0.80 (−0.26 to 1.87)	.139
12 months	21.68 (0.51)	−0.66 (−1.61 to 0.30)	142	21.72 (0.47)	−0.90 (−1.55 to −0.24)	146	0.24 (−0.92 to 1.40)	.688

## Discussion

In this randomized controlled trial, a 1-year combined PTCT program did not produce additional benefits for fall prevention when compared with multicomponent physical exercise alone among community-dwelling older adults who did not have cognitive impairments and did not meet the physical activity guidelines prior to the intervention. PTCT reduced concern about falling to a similar extent as PT alone among these relatively well-functioning older people.

The combination of PTCT has attracted attention recently, but its effects on falls have not been widely published. Two small studies using variate statistical approaches to analyze the effects of intervention on falls among older people without cognitive impairment (35) and with mild cognitive impairment (17) have reported that physical exercise with simultaneously performed cognitive tasks did not produce enhanced value for fall prevention when compared with PT alone. In our study, the PTCT exercises were organized separately. In line with those earlier studies, the incidence of falls was comparable in both study groups during the 1-year intervention and 1-year postintervention follow-up.

Although the PT with separate CT had no additional effects on the rate of falls compared with intensive multicomponent PT alone, it improved the relevant risk factor for falls and executive functions. We have previously shown that both groups improved their executive functions, but the combined training induced two- to threefold greater improvements (15). It has been proposed that executive functions are important cognitive components of walking and are related to better performance, in particular under challenging walking conditions (36). Moreover, improved executive functions may promote the maintenance of the level of physical activity in the long term (37). Through these 2 mechanisms, the beneficial effects on executive functions could result in delayed effects in terms of fall prevention. Thus, it would be beneficial to follow up on falls even more than 1 year after the intervention.

Systematic review evidence from 59 trials has indicated that physical exercise as a single intervention has a moderate (23%) effect in preventing falls when compared with the control conditions, which are not perceived as reducing falls (8). In the current study, we were unable to assess the effects of PT alone on falls because of the lack of an untrained control group. However, the rate of falls per person-years (IR 1.06, 95% CI 0.85–1.34) among the participants in the PT alone group in our study was comparable to that of the other trials involving physical exercise groups, and it was lower than that in the control groups reported earlier in studies with comparable populations (38–40). Thus, our study supports earlier findings that exercising according to physical activity recommendations is beneficial for fall prevention.

Among these relatively healthy older adults, the average baseline level of concern about falling was moderate based on the cutoff scores (low 16–19, moderate 20–27, and high concern 28–64) published by Delbaere et al. (6). Concern about falling reduced slightly but similarly in both groups during the 1-year intervention. This is not surprising because the walking and executive functions improved significantly in both groups, as previously reported (15). Improved walking capacity and executive functions have been shown to enhance fall-related self-efficacy (41). Randomized controlled trials using FES-I have reported decreases in scores ranging from 0.53 to 3.7 (9). The results of our study fell within this range. Thus, PT with and without the CT component reduced concern about falling to a small but comparable degree immediately after the intervention. A longer follow-up is warranted because lower concern about falling has previously been shown to be associated with less prospective or recurrence of falls in older adults (42).

The current study has several strengths but also limitations. The study population comprised a representative sample of community-dwelling 70- to 85-year-old people who did not meet physical activity recommendations prior to the study. Thus, generalization is restricted to older adults not meeting physical activity recommendations. Our results cannot be extrapolated to older adults with a high risk of falling because of physical and/or cognitive impairments. The falls were ascertained monthly from diaries, but the participant-reported injurious falls were not verified from medical records. The present RCT involved feasible long-term training interventions that achieved relatively high adherence. The PT program followed the physical activity guidelines of the time, which have proven effective in reducing the number of falls in older adults. The attrition rate was low. Sample size calculation was based on a 27% lower rate of falls in the PTCT compared with the PT group, here requiring 155 participants per group. The fall rate estimate due to the PT intervention was based on the data from Uusi-Rasi et al. (32). It is a limitation that an estimate of the effect of a CT was not available to us to be used in our power analysis at the time of planning this study. We observed a 22% difference between the PTCT and PT in the fall rate. Thus, the observed IRR was somewhat lower than the effect size estimate used in our power calculation.

In conclusion, a yearlong PTCT intervention did not result in a significantly lower rate of falls or injurious falls than PT alone in older community-dwelling men and women who did not meet the physical activity guidelines prior to the intervention. In addition, combined training reduced concern about falling to a similar extent as PT alone among these relatively healthy older adults.
